# 

**Published:** 1894-05

**Authors:** 


					﻿Vol xiv.
MAY, 1894.
No. 5,
THE
pOMIOPATHlC pHY^lClAfl,
A Monthly Journal of Homœopathic Materia Medica E
and Clinical Medicine.
“ He it a freeman whom truth majcet free, and all are tlavet betide."
“Seek the Truth: come whence it may, cost what it will."
ISltn AND PUBLISHED BY
WALTER M. JAMES, M. D.
PHILADELPHIA :
No. lies SPRUCE STREET.
LONDON:
ALFRED HEATH & CO., Sons Agents bob Gbeat Bbitain,
114 Ebury Street, Eaton Square.
•Yearly Subscription, $2.50, invariably in advance. Single number t, 95 etc.
Entered at the PbUad.lphla Paat-Ottee M Seoaod-Oaaa Mall Matter.
				

